# Prevalence and clinical profiles of *Mycoplasma haemofelis* and ‘Candidatus* Mycoplasma haemominutum*’ in domestic cats in Southern Vietnam

**DOI:** 10.5455/javar.2025.l990

**Published:** 2025-12-25

**Authors:** Thao Tran Thi, Chien Tran Phuoc Nguyen, Dac Gia Luu, Tham Thi Dang, Bich Tran Ngoc

**Affiliations:** Faculty of Veterinary Medicine, College of Agriculture, Can Tho University, Can Tho, Vietnam

**Keywords:** *Mycoplasma haemofelis*, Candidatus *Mycoplasma haemominutum*, PCR, *16S rRNA* gene, Vietnam

## Abstract

**Objectives:**

This study aimed to investigate the molecular prevalence and species distribution of feline hemoplasmas—*Mycoplasma haemofelis* (Mhf) and “Candidatus *Mycoplasma haemominutum”* (CMhm)—in domestic cats in southern Vietnam. The *16S rRNA* gene was used as a molecular marker for this research. To assess their clinical relevance, the blood and chemical profiles of the infected cats were also examined.

**Materials and Methods:**

We collected a total of 68 blood samples from domestic cats brought to the Veterinary Teaching Clinic, Faculty of Veterinary Medicine, Can Tho University, Vietnam. To screen for hemoplasma, genomic deoxyribonucleic acid was extracted, and conventional polymerase chain reaction (PCR) was used to target the *16S rRNA* gene. We sequenced the positive samples and then used nucleotide BLAST to identify the species. The simulated hematological and biochemical data, including hematocrit, alanine aminotransferase (ALT), and blood urea nitrogen (BUN), were reviewed to support clinical interpretation.

**Results:**

In this study, five of the 68 samples (7.35%) tested positive for feline hemoplasmas. Two of the samples were identified as *M. haemofelis*, while three were classified as “Candidatus *Mycoplasma haemominutum”*. Cats infected with Mhf exhibited normocytic normochromic anemia, accompanied by increased ALT levels and a slight elevation in BUN. These findings suggest that the infection affects the whole body. In contrast, cats that tested positive for CMhm showed no clinical symptoms, and their blood and biochemical tests were normal.

**Conclusion:**

This study confirms the presence of both Mhf and CMhm in domestic cats in Southern Vietnam. Although CMhm is generally considered low virulence, the hematobiochemical alterations observed in Mhf-positive cats warrant attention. PCR targeting the *16S rRNA* gene proves effective for diagnosis and epidemiological surveillance.

## Introduction

Hemotropic mycoplasmas, also known as hemoplasmas, are parasitic bacteria that lack cell walls. These organisms, which belong to the Mollicutes class, cannot be cultivated in a laboratory. These pathogens can be found in the blood of mammals, including domestic animals like cats and dogs. Hemoplasmas cause infections by attaching to and multiplying on the surfaces of red blood cells [1]. These small, cell wall-deficient, epierythrocytic bacteria—*Mycoplasma haemofelis* (Mhf), “Candidatus *Mycoplasma haemominutum*” (CMhm), and “Candidatus *Mycoplasma turicensis*”—are obligate red blood cell parasites capable of inducing a spectrum of clinical outcomes, ranging from asymptomatic carriage to life-threatening hemolytic anemia [2,3]. Among these, *M*. *haemofelis* is known to be the most harmful species, often causing noticeable anemia. In contrast, CMhm is generally considered less harmful and is often found in individuals who exhibit no symptoms or are infected with multiple pathogens [2].

Despite their global presence, Southeast Asia lacks comprehensive epidemiological data, particularly regarding the frequency of different species, their clinical associations, and molecular confirmation. Previous studies have shown that the prevalence of this condition varies significantly, depending on the study location, the type of cat population being examined (including both pet and feral cats), and the diagnostic methods used. The global prevalence of CMhm varies widely, ranging from 4.4% to 46.7%, while Mhf shows a range from 0.4% to 27.0% [4]. A pooled meta-analysis conducted across five Southeast Asian nations—Indonesia, Malaysia, the Philippines, Taiwan, and Thailand—revealed an overall prevalence of feline hemoplasma at 16.13%. Within this, CMhm and Mhf were identified in 8.6% and 6.45% of the cases, respectively [5]. In Bangkok, Thailand, a study of semi-feral cats showed a 38.05% infection rate for hemoplasma. Of the positive cases that were sequenced, 83.33% were identified as CMhm [6]. In contrast, other research in Thailand found a 25% prevalence of the infection in domestic cats [7]. In contrast, Malaysian data showed a notably high prevalence of Mhf, reaching 68.8% [8]. In comparison, studies from China and Turkey reported much lower rates, ranging from 0.9% to 3.4% and 0.81% to 8.13%, respectively [9,10]. These differences reflect the complex epidemiological patterns of feline hemoplasmas, which are influenced by local vector populations, the availability of diagnostic tools, and the manner in which cats are cared for.

Molecular diagnostics, particularly polymerase chain reaction (PCR) methods targeting the *16S rRNA* gene, have demonstrated significantly greater sensitivity than traditional cytology for identifying hemoplasmas [11,12]. Although the *16S rRNA* gene is widely used to determine evolutionary relationships between species, it can be difficult to distinguish closely related strains. This is because additional gene targets, such as the *23S rRNA* gene, are often required [12].

Conversely, the limited availability of molecular epidemiological data in Vietnam presents a diagnostic and therapeutic challenge for veterinarians, thereby impeding evidence-based decision-making processes and the formulation of targeted control strategies. Determining the local prevalence and clinical characteristics of Mhf and CMhm is essential for improving the health of cats. Therefore, this study aimed to investigate the molecular prevalence and species distribution of feline hemoplasmas, particularly *M*. *haemofelis* and CMhm, which were identified in domestic cats brought to the Veterinary Teaching Clinic at Can Tho University in Vietnam. To understand the molecular characteristics and clinical effects of hemoplasma infections in cats from the Mekong Delta, we employed *16S rRNA* PCR detection in conjunction with blood tests and biochemical analyses.

## Materials and Methods

### Ethical clearance

This research was conducted on animals with naturally occurring infections. All samples used in this study were diagnostic samples collected as part of routine clinical examination, and no experimental interventions were performed on the animals. Blood sample collection was carried out in strict compliance with the national technical regulation QCVN 01-83:2011/BNNPTNT on Animal Diseases—General Requirements for Sample Collection, Storage, and Transportation issued by the Ministry of Agriculture and Rural Development of Vietnam. All experimental procedures were reviewed and approved by the Institutional Animal Care and the Animal Ethics Committee of Can Tho University, Vietnam [Approval No. CTU-AEC2401, Approval Date: 26 June 2024]. Informed consent was obtained from all animal owners prior to sample collection.

### Sample collection and clinical data

Between January and June 2025, 68 client-owned domestic cats presenting to the Veterinary Teaching Clinic (Faculty of Veterinary Medicine, Can Tho University, Vietnam) were randomly selected from daily appointments and screened for feline hemoplasmas by *16S rRNA* PCR, irrespective of the presenting complaint. Signalment (age, sex, breed, husbandry) and standardized clinical findings were recorded. For descriptive analyses, cats were classified as symptomatic (≥ 1 sign compatible with hemoplasmas: pale mucous membranes, fever, lethargy, anorexia, lymphadenopathy) or asymptomatic.

Whole blood was obtained via cephalic or jugular venipuncture into EDTA tubes using aseptic technique. Samples were stored at 4°C and processed for deoxyribonucleic acid (DNA) extraction within 24 h of collection. Hematological analysis was performed using an automated veterinary hematology analyzer (DREW SCIENTIFIC Excell 2280, USA), and parameters were compared with established reference intervals for healthy domestic cats [13].

For all PCR-positive cats, complete blood counts were evaluated, including hematocrit (HCT), red blood cell count (RBC), hemoglobin concentration (HGB), mean corpuscular volume (MCV), mean corpuscular hemoglobin concentration (MCHC), total white blood cell count (WBC), monocyte count, and platelet count (PLT). Serum biochemical analyses were performed using a biochemistry analyzer (BSI 3000 Evolution, Italy). All measurements were interpreted according to established feline reference intervals [13].

## DNA extraction and PCR amplification

The TopPURE^®^ Viral DNA/RNA Extraction Kit (ABT, Vietnam) was used to extract genomic DNA from 200 µl of EDTA-anticoagulated whole blood, following the manufacturer’s instructions. A standard PCR assay targeting a conserved region of the 16S rRNA gene of hemoplasmas was employed, using primers previously described by Criado-Fornelio et al. [14]. The forward primer (HBT-F) was 5’-ATA CGG CCC ATA TTC CTA CG-3’ (positions 313–332 in GenBank AF178677), and the reverse primer (HBT-R) was 5’-TGC TCC ACC ACT TGT TCA-3’ (positions 889–908 in AF178677). This should have made an amplicon of about 595 bp.

In each 25 µl PCR reaction mixture, there were 12.5 µl of 2 × PCR Master Mix (Bioline, UK), 1 µl of each primer (10 µM), 2 µl of extracted DNA template, and 8.5 µl of water that did not contain nucleases. The thermocycling conditions started with denaturation at 95°C for 10 min. Then, there were 35 cycles of denaturation at 95°C for 30 sec, annealing at 58°C for 30 sec, and extension at 72°C for 30 sec. The last step was elongation at 72°C for 5 min. Electrophoresis was used to visualize amplification products on a 1.5% agarose gel stained with GelGreen (ABT, Vietnam) and illuminated by UV light.

### Sequencing and phylogenetic analysis

We used the TopPure^®^ PCR/Gel Purification Kit (ABT, Vietnam) to clean up the PCR-positive amplicons and then sent them to NamKhoa Biotech (Vietnam) for bidirectional Sanger sequencing. We used BioEdit version 7.5.2 to assemble and manually edit the raw chromatograms. BLASTn (https://blast.ncbi.nlm.nih.gov) was used to determine sequence identity by comparing the sequence to validated reference sequences in GenBank. The species of hemoplasma was determined by comparing it to type strains with at least 97% nucleotide identity.

Using ClustalW in MEGA version 12 [15], assembled sequences were lined up with representative hemoplasma sequences from GenBank. We used the Maximum Likelihood method in MEGA version 12, with the Tamura–Nei substitution model, to determine phylogenetic relationships. We used 1,000 bootstrap replicates to check how well the branches supported each other. The phylogenetic tree generated from this analysis was used to confirm clustering at the species level and to examine the degree of relatedness between local isolates and global reference strains.

## Results

### Clinical and demographic findings

At presentation, 9/68 (13.2%) were symptomatic and 59/68 (86.8%) asymptomatic. Table 1 summarizes PCR outcomes by clinical status with exact (Clopper–Pearson) 95% CIs; Table 2 lists signalment and clinical features of PCR-positive cats. Among 68 cats, five tested Positive for feline hemoplasmas, yielding an overall prevalence of 7.35% (95% CI 2.43–16.33). Species-level results were *M*. *haemofelis* 2/68 = 2.94% (95% CI 0.36–10.22) and “Candidatus *M. haemominutum*” 3/68 = 4.41% (95% CI 0.92–12.36) (Table 1). Sequencing of the *16S rRNA* gene identified two Mhf and three “Candidatus* Mycoplasma haemominutum*” (CMhm) infections (Table 2). Mhf-positive cats (CT-M21 and CT-M22) were presented with clinical signs including lethargy, pale mucous membranes, disorientation, and anemia. CMhm-positive cats (CT-M18, CT-M32, and CT-M45) were asymptomatic or identified incidentally during routine screening.

**Table 1.** Distribution of Mhf and CMhm infection status and clinical signs in examined animals.

Observation	PCR positive (*n*, % [95%CI])	PCR negative (*n* , % [95%CI])
Symptomatic	2 (2.94%) [95%CI: 0.36–10.22]	7 (10.29%) [95%CI: 4.24–20.07]
Asymptomatic	3 (4.41%) [95%CI: 0.92–12.36]	56 (82.35%) [95%CI: 71.20–90.53]
Total	5 (7.35%) [95%CI: 2.43-16.33]	63 (92.65%) [95%CI: 83.67–97.57]

Note: Exact (Clopper–Pearson) 95% binomial confidence intervals are reported. Totals: *n* = 68 (Positive 5, Negative 63). Mhf, *Mycoplasma haemofelis*; CMhm, ‘Candidatus *Mycoplasma haemominutum*’.

**Table 2.** Clinical and demographic data of feline hemoplasma-positive cases.

Cat ID	Location	Breed	Age (months)	Sex	Husbandry	Species	Clinical signs
CT-M21	Phong Dien	Domestic	24	Female	Free roaming	Mhf	Lethargy, pale mucous membranes, mild fever
CT-M22	Ninh Kieu	British Shorthair	36	Male	Indoor	Mhf	Disorientation, weakness, severe anemia
CT-M18	Cai Rang	Domestic	48	Female	Free roaming	CMhm	No observable clinical signs
CT-M32	Cai Rang	British Longhair	12	Male	Indoor	CMhm	Incidental finding
CT-M45	Ninh Kieu	Persian	30	Female	Indoor	CMhm	No observable clinical signs

Note: Mhf, *Mycoplasma haemofelis*; CMhm, Candidatus *Mycoplasma haemominutum*. Only CT-M21 and CT-M22 showed clinical signs; the remaining three positive cases were asymptomatic.

### Hematological findings

Both *M*. *haemofelis*-infected cats (CT-M21 and CT-M22) demonstrated hematological alterations consistent with regenerative anemia. Hematocrit values were markedly reduced (18% and 15%), accompanied by decreased RBC counts (3.5 and 3.1 ×10⁶/µl) and HGB concentrations (6.2 and 5.8 gm/dl) (Table 3). Mean corpuscular volume and MCHC remain within reference intervals, indicating a normocytic, normochromic anemia pattern characteristic of immune-mediated hemolysis associated with *M*. *haemofelis*.

**Table 3.** Hematological profiles of Mhf- and CMhm-infected cats.

Parameter	Reference Range^*^	CT-M21 (Mhf)	CT-M22 (Mhf)	CT-M18 (CMhm)	CT-M32 (CMhm)	CT-M45 (CMhm)
RBC (×10⁶/µl)	5–10	3.5↓	3.1↓	6.0	6.2	5.8
HCT (%)	30–45	18↓	15↓	32	34	30
HGB (gm/dl)	8–15	6.2↓	5.8↓	9.8	10.1	9.5
MCV (fl)	39–55	51.4	48.4	53.3	54.8	51.7
MCHC (gm/dl)	30–36	34.4	33.7	32.4	32.7	31.6
WBC (×10³/µl)	5.5–19.5	12.0↑	14.5↑	9.0	8.7	9.5
Neutrophils (×10³/µl)	2.5–12.5	8.1	9.4	5.2	5.0	5.4
Lymphocytes (×10³/µl)	1.5–7	3.2	4.5	3.1	2.8	3.0
Monocytes (×10³/µl)	0–0.85	0.9↑	1.0↑	0.6	0.5	0.4
PLT (×10³/µl)	200–500	180↓	135↓	240	260	250

Note: Mhf, *Mycoplasma haemofelis*; CMhm, Candidatus *Mycoplasma haemominutum*. RBC = red blood cells; HCT = hematocrit; HGB = hemoglobin; MCV = mean corpuscular volume; MCHC = mean corpuscular hemoglobin concentration; WBC = white blood cells; PLT = platelets.

Leukocytosis was present in both cases (12.0 and 14.5 × 10³/µl), primarily driven by monocytosis (0.9 and 1.0 × 10³/µl), suggesting chronic inflammatory stimulation and active macrophage-mediated erythrophagocytosis. Mild thrombocytopenia was also observed (180 and 135 × 10³/µl), potentially reflecting immune-mediated platelet destruction, consumptive coagulopathy, or splenic sequestration secondary to systemic inflammation.

In contrast, all three CMhm-infected cats (CT-M18, CT-M32, and CT-M45) exhibited hematological parameters within normal physiological limits. These cats did not display anemia, leukocytosis, or thrombocytopenia, and their differential leukocyte counts remained within reference ranges. This absence of hematological abnormalities reinforces the classification of CMhm as a low-virulence hemoplasma in immunocompetent hosts, typically associated with subclinical carriage rather than overt clinical disease.

***Biochemical findings***

The two *M*.* haemofelis*-infected cats (CT-M21 and CT-M22) showed slightly higher globulin levels (4.8 and 5.6 gm/dl) and increased total protein. These findings suggest a prolonged immune response to an antigen and a general inflammatory reaction in the body. In both cases, aspartate aminotransferase (AST) activity was slightly elevated, measuring 72 and 85 U/l. This could indicate liver damage caused by a systemic illness or muscle breakdown. Notably, the total bilirubin levels increased, measuring 0.8 and 1.1 mg/dl. This finding is consistent with pre-hepatic hyperbilirubinemia, a common result of *M. haemofelis* infection, caused by hemolysis. Table 4 shows the destruction of red blood cells caused by *Haemofelis*.

**Table 4.** Serum biochemical profiles of feline hemoplasma-positive cats.

Parameter	Reference Range ^ *^	CT-M21 (Mhf)	CT-M22 (Mhf)	CT-M18 (CMhm)	CT-M32 (CMhm)	CT-M45 (CMhm)
Albumin (gm/dl)	2.5–4.0	3.0	2.6	3.2	3.1	3.3
Globulin (gm/dl)	2.5–5.0	4.8	5.6↑	3.3	3.6	3.1
ALT (U/l)	20–100	88	95	42	51	46
AST (U/l)	10–60	72↑	85↑	39	44	41
BUN (mg/dl)	15–34	28	30	25	26	24
Creatinine (mg/dl)	0.8–2.4	1.6	1.8	1.2	1.4	1.3
Total Bilirubin (mg/dl)	0.0–0.4	0.8↑	1.1↑	0.3	0.2	0.2

Note: Mhf, *Mycoplasma haemofelis*; CMhm, Candidatus *Mycoplasma haemominutum*. AST = aspartate aminotransferase; ALT = alanine aminotransferase; BUN = blood urea nitrogen (urea). ^*^Species-specific reference intervals (Merck Veterinary Manual, 2013, 11th ed.).

^*^Species-specific reference intervals (Merck Veterinary Manual, 2013, 11th ed.).

In contrast, all the cats infected with CMhm (CT-M18, CT-M32, CT-M45) showed serum biochemical values within the normal range, indicating that their liver and kidneys were functioning properly. The absence of biochemical abnormalities supports the notion that CMhm infection is subclinical in otherwise healthy cats with normal immune systems, and it does not appear to affect their organs.

### Phylogenetic analysis

**Figure 1.**
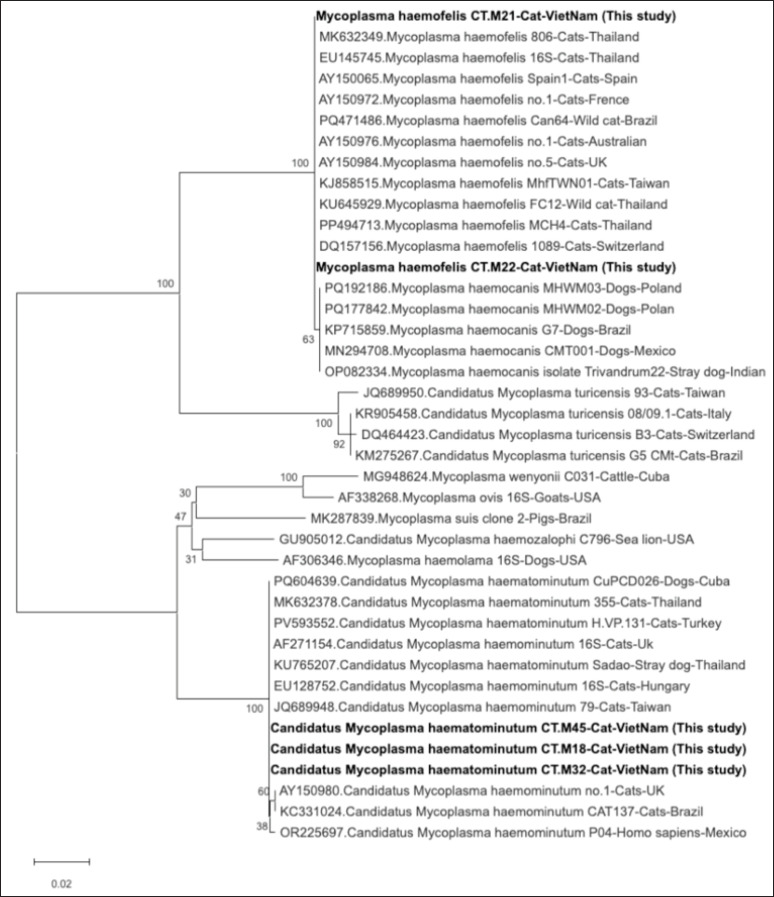
Maximum likelihood phylogenetic tree of partial *16S rRNA* sequences from feline hemoplasmas detected in this study and reference sequences from GenBank. The tree was constructed using the maximum likelihood method with 1,000 bootstrap replicates; Vietnamese isolates are indicated in bold; Bootstrap values ≥ 70% are shown at the nodes; Scale bar indicates the number of nucleotide substitutions per site.

Maximum likelihood analysis of partial *16S rRNA* sequences revealed three well-supported clades corresponding to Mhf and CMhm (Fig. 1). The two Vietnamese Mhf isolates (CT.M21, CT.M22) clustered tightly with reference Mhf strains from cats in Thailand, Brazil, France, Australia, Taiwan, and Switzerland (bootstrap = 100), indicating high genetic conservation. These isolates shared 99.65% sequence identity with Mhf previously detected in stray dogs in India (OP082334) and in domestic dogs from Poland (PQ192186, PQ177842), Brazil (KP715859), and Mexico (MN294708). All three CMhm isolates (CT.M45, CT.M18, and CT.M32) grouped together within the CMhm clade, along with sequences from cats in Thailand, Brazil, Turkey, Hungary, and the UK (bootstrap = 100). They shared 99.49%–99.83% identity with CMhm sequences reported from stray dogs in Thailand (KU765207) and domestic dogs in Cuba (PQ604639). These results confirm the co-circulation of pathogenic Mhf and low-virulence CMhm in southern Vietnam, both of which show close genetic relatedness to strains reported globally.

## Discussion

This study provides the first molecular evidence of co-circulation of Mhf and “Candidatus* Mycoplasma haemominutum*” (CMhm) in domestic cats from Southern Vietnam. This study analyzed a random sample of 68 client-owned cats presented to the Can Tho University Veterinary Teaching Clinic and documented an overall hemoplasma prevalence of 7.35% (Mhf and CMhm). This rate is lower than in five countries (Indonesia, Malaysia, the Philippines, Taiwan, and Thailand), which reported an overall feline hemoplasma prevalence of 16.13%, with CMhm and Mhf accounting for 8.6% and 6.45%, respectively [3,5,8], but higher than some European surveys (< 5%) [2]. The observed differences may reflect variation in vector abundance, cat management practices, and sampling strategies.

Cats infected with Mhf exhibited clear signs of illness, as indicated by blood tests that revealed anemia with a regenerative pattern, normal-sized and normal-colored red blood cells, an increase in monocytes, and a slight decrease in platelets. These observed abnormalities are consistent with the destruction of red blood cells by the immune system and the activation of macrophages in the spleen, which is a key feature of Mhf disease development [3,16,17]. In these cases, the high bilirubin levels likely indicate a buildup of bilirubin before it reaches the liver, caused by the breakdown of red blood cells within the blood vessels. At the same time, the increased AST levels might be due to damage to both liver cells and muscle tissue, which is related to the overall illness. In contrast, cats with CMhm showed no clinical signs of illness. Their blood tests and serum biochemistry were normal, which supports the idea that it is a less harmful type of hemoplasma in cats with healthy immune systems [1,2,6].

Demographically, both Mhf and CMhm infections occurred in purebred and domestic shorthair cats; however, Mhf cases included one free-roaming cat, which aligns with the known role of ectoparasites, such as fleas (*Ctenocephalides felis*) and ticks (*Rhipicephalus sanguineus*), in the transmission of hemoplasmas [2,18, 19]. Indoor confinement in CMhm-positive cats may reduce vector exposure, contributing to the absence of clinical signs. However, the detection of CMhm in strictly indoor cats suggests that indirect transmission via transient vector introduction or prior exposure before adoption cannot be excluded. Although our cohort included indoor-only cats, intermittent household exposure to *Ctenocephalides felis* cannot be excluded and remains the most plausible route of acquisition [18,20,21]. Rare iatrogenic transmission via blood products or needle reuse has also been postulated, and some cats may have acquired infection before adoption and maintained low-level bacteremia. These mechanisms likely explain CMhm detection in cats that do not have access to the outdoors.

Phylogenetic analysis of *16S rRNA* sequences revealed three distinct clades, corresponding to Mhf, CMhm, and CMt. This finding is consistent with existing global classification systems [4,6,12]. The Vietnamese Mhf isolates showed genetic similarity to strains from various continents. This suggests a high level of sequence conservation, indicating a stable global lineage. In contrast, CMhm sequences showed slightly more genetic variation, but they still belonged to the CMhm group. The CMhm isolates from this study showed a 99.49%–99.83% similarity to CMhm found in stray dogs in Thailand (GenBank accession no. KU765207). This suggests the possibility of transmission between species or shared environmental sources in Southeast Asia [5-7]. These findings underscore the potential for hemoplasma transmission between species, facilitated by shared ectoparasite vectors and the overlapping urban environments of cats and dogs [23].

From a clinical perspective, PCR testing, which targets the hemoplasma *16S rRNA* gene, is a better diagnostic method than cytology [11,14,22]. When *M*. *Haemofelis* infection is suspected or confirmed, the initial treatment is doxycycline, administered orally at a dose of approximately 10 mg/kg per day for 21–28 days [24]. Glucocorticoids, given for a short time, might be used only if there’s a strong suspicion of immune-mediated hemolysis. All cats that are in contact with each other should be treated with effective ectoparasite control methods, such as isoxazolines [25]. Monitoring packed-cell volume/hematocrit during and after treatment is crucial for identifying potential relapses [22].

From a veterinary perspective, the presence of both a highly pathogenic species (Mhf) and a less pathogenic species (CMhm) in the same area requires species-specific molecular tests to inform clinical decisions. Misdiagnosis could lead to either unnecessary treatments for mild infections or inadequate care for more serious cases. From an epidemiological standpoint, these results underscore the need for integrated vector control programs that encompass both cats and dogs, particularly in areas where animals are allowed to roam freely. The study’s limitations include its single-center, clinic-based sampling method, a relatively small sample size, and the lack of systematic testing for co-infections, such as FeLV/FIV, as well as vector assessments. These limitations could potentially skew prevalence estimates, either upwards or downwards, and therefore require confirmation through larger, multi-center studies.

## Conclusion

This study confirms the first detection of Mhf and “Candidatus *Mycoplasma haemominutum*” in domestic cats in Southern Vietnam at an overall prevalence of 7.35% (95% CI 2.43–16.33). Phylogenetic analysis revealed high genetic similarity with regional strains*. Mycoplasma* *haemofelis*-positive cats exhibited normocytic normochromic anemia with mild increases in ALT/BUN, whereas CMhm-positive cats were clinically unremarkable. Our findings underscore the value of PCR for diagnosis and routine flea control in feline practice in the region, motivating larger, multi-center studies that incorporate vector assessment and co-infection testing.

## Data Availability

De-identified data and the analysis code (R Studio) used to compute exact (Clopper–Pearson) 95% confidence intervals are available from the corresponding author upon reasonable request, subject to institutional and ethical approvals. All aggregated results are reported in the tables. No sequence data were generated.

## List of abbreviations

ALT, alanine aminotransferase; AST, aspartate aminotransferase; BLASTn, Basic Local Alignment Search Tool (nucleotide); bp, base pair(s); BUN, blood urea nitrogen; CI, confidence interval (95% CI); CMhm, ‘Candidatus* Mycoplasma haemominutum*’; CMt, ‘Candidatus* Mycoplasma turicensis*’; DNA, deoxyribonucleic acid; EDTA, ethylenediaminetetraacetic acid; HCT, hematocrit; HGB, hemoglobin; MCHC, mean corpuscular hemoglobin concentration; MCV, mean corpuscular volume; MEGA, Molecular Evolutionary Genetics Analysis; Mhf, *Mycoplasma haemofelis*; ML, maximum likelihood; PCV, packed cell volume; PCR, polymerase chain reaction; PLT, platelet count; RBC, red blood cell (count); RNA, ribonucleic acid; WBC, white blood cell (count); gm, grams; kg, kilograms; mg, milligrams; h, hours; sec, seconds; µl, microliter.
